# Wake slow waves and aperiodic activity during resting‐state EEG correlate with amyloid burden and hypometabolism

**DOI:** 10.1002/alz.085898

**Published:** 2025-01-09

**Authors:** Pierre Champetier, Claudia Albero, Filipa Raposo Pereira, Rubén Herzog, Maximilien Chaumon, Marion Houot, Nathalie George, Isabelle Arnulf, Delphine Oudiette, Thomas Andrillon, INSIGHT‐preAD group

**Affiliations:** ^1^ Sorbonne Université, Paris Brain Institute (ICM), INSERM, CNRS, UMR‐1127, Mov’It, DreamTeam, Paris France; ^2^ AP‐HP, Hôpital Pitié‐Salpêtrière, Service des Pathologies du Sommeil, National Reference Center for Narcolepsy, Paris France; ^3^ Sorbonne Université, Paris Brain Institute (ICM), INSERM, CNRS, CENIR MEG‐EEG, Paris France; ^4^ Institut de la Mémoire et de la Maladie d’Alzheimer (IM2A), Départment de Neurologie, Paris France

## Abstract

**Background:**

Spectral power of slow rhythms in resting‐state EEG increases along Alzheimer's disease (AD) continuum. Besides, recent studies have revealed 1) the importance of analyzing the aperiodic component of an EEG power spectrum and 2) the intrusions of sleep‐like slow waves identifiable in wake EEG of animals and young adults. Importantly, the occurrence of these wake slow waves is known i) to increase after sleep deprivation, ii) to be associated with markers of sleepiness, and iii) to predict behavioral errors at different tasks. Our objective was to investigate the links between AD biomarkers and 1) the aperiodic component of the EEG signal and 2) wake slow waves.

**Method:**

314 elderly individuals with memory complaints from the INSIGHT‐preAD cohort (76.5 ± 3.5 years, range: 70‐85 years) were recruited. Participants underwent a 5‐year follow‐up, including 1) high‐density EEG recordings (256 electrodes) at rest (2 sessions of 2 min) each year, 2) neuropsychological assessments each year, and 3) Florbetapir and ^18^F‐FDG PET scans at baseline to quantify amyloid burden and hypometabolism. For each EEG recording, characteristics of aperiodic activity (intercept and slope) and wake slow waves (density, amplitude, slope, and frequency) were determined. For each neuroimaging modality, mean standardized uptake value ratios (SUVRs) were computed in AD relevant brain regions. Associations between AD biomarkers (amyloid and FDG SUVRs) and EEG metrics (characteristics of aperiodic activity and slow waves) were tested using linear regressions controlled for age, sex, and education level, after FDR correction. Longitudinal effects, including neuropsychological metrics, will be tested using linear mixed effect models.

**Result:**

At baseline, high amyloid burden was positively associated with 1) the intercept and slope of aperiodic activity and 2) the density and frequency of slow waves. Besides, hypometabolism was associated with lower slow wave density.

**Conclusion:**

These preliminary results suggest that aperiodic activity and wake slow waves could serve as early EEG indicators of certain AD biomarkers. This seems particularly promising as these metrics are quantifiable on a simple resting EEG and at a very early stage, even before the onset of cognitive alterations. Longitudinal and neuropsychological data will be analyzed in the next few months.

